# Expression patterns of microRNAs associated with CML phases and their disease related targets

**DOI:** 10.1186/1476-4598-10-41

**Published:** 2011-04-18

**Authors:** Kateřina Machová Poláková, Tereza Lopotová, Hana Klamová, Pavel Burda, Marek Trněný, Tomáš Stopka, Jana Moravcová

**Affiliations:** 1 Institute of Hematology and Blood Transfusion, Prague, U Nemocnice 1, 128 20, Czech Republic; 2 The Faculty of Science, Charles University, Prague, Viničná 5, 128 00, Czech Republic; 3 First Faculty of Medicine and Center of Experimental Hematology, Charles University in Prague, U Nemocnice 5, 128 53, Czech Republic

## Abstract

**Background:**

MicroRNAs are important regulators of transcription in hematopoiesis. Their expression deregulations were described in association with pathogenesis of some hematological malignancies. This study provides integrated microRNA expression profiling at different phases of chronic myeloid leukemia (CML) with the aim to identify microRNAs associated with CML pathogenesis. The functions of *in silico *filtered targets are in this report annotated and discussed in relation to CML pathogenesis.

**Results:**

Using microarrays we identified differential expression profiles of 49 miRNAs in CML patients at diagnosis, in hematological relapse, therapy failure, blast crisis and major molecular response. The expression deregulation of miR-150, miR-20a, miR-17, miR-19a, miR-103, miR-144, miR-155, miR-181a, miR-221 and miR-222 in CML was confirmed by real-time quantitative PCR. *In silico *analyses identified targeted genes of these miRNAs encoding proteins that are involved in cell cycle and growth regulation as well as several key signaling pathways such as of mitogen activated kinase-like protein (MAPK), epidermal growth factor receptor (EGFR, ERBB), transforming growth factor beta (TGFB1) and tumor protein p53 that are all related to CML. Decreased levels of miR-150 were detected in patients at diagnosis, in blast crisis and 67% of hematological relapses and showed significant negative correlation with miR-150 proved target *MYB *and with *BCR-ABL *transcript level.

**Conclusions:**

This study uncovers microRNAs that are potentially involved in CML and the annotated functions of *in silico *filtered targets of selected miRNAs outline mechanisms whereby microRNAs may be involved in CML pathogenesis.

## Introduction

Mammalian microRNAs (miRNA, miR) are short non-coding RNAs that regulate preferentially gene expression by inhibiting translation of specific target mRNAs. MiRNA-mRNA matching is based on imperfect sequence base-pairing with the required complementarity centered over positions 2 - 8 of mRNA's seed sequence [1]. Depending on specific target genes, miRNAs regulate many cellular functions such as developmental timing, signal transduction, apoptosis, cell proliferation and tumorigenesis [2-5]. Thus, gene expression and role of miRNAs are currently being largely studied in human malignancies and chemical compounds that regulate miRNA levels are potentially very important for developing new treatment strategies in chronic myeloid leukemia (CML). The first miRNA molecules that have been associated with human leukemia pathogenesis were found in chronic lymphocytic leukemia (CLL) [6]. MiR-15 and miR-16 are located in a genomic region that is frequently deleted in CLL, thus the expression of these two miRNAs is downregulated. Other works brought the evidence that many miRNAs are indeed found at chromosomal breakpoints and genomic regions associated with cancer [7,8].

In CML the following miRNAs were associated with the disease pathogenesis. For instance, the miR-203 was found to be epigenetically silenced in human leukemic Philadelphia chromosome-positive (Ph+) cell lines; this is in line with the observation that BCR-ABL and ABL kinases are miR-203 putative targets [9]. Derivative 9q+ chromosome deletions carrying miR-199b that occurred in some CML patients were associated with miR-199b decrease [10]. Venturini et al. [11] showed miR-17-92 cluster (onkomir-1) to be aberrantly expressed in CD34+ cells of CML patients. Agirre at al. [12] analyzed the expression of 157 miRNAs in mononuclear and CD34+ cells separated from bone marrow of 6 CML patients at diagnosis and found 11 miRNAs (e.g. miR-150, miR-151, miR-221, miR-127, miR-16) aberrantly expressed in CD34+ cells and 53 miRNAs differentially expressed in mononuclear cells (e.g. miR-150, miR-126, miR-221, miR-222, miR-21). Two recent works contributed to the knowledge about expression change in specific microRNAs associated with resistance to imatinib or responsiveness to imatinib after the treatment initiation in CML patients [13,14]. A group of 19 miRNAs (e.g. miR-191, miR-29a, miR-422b, miR-100, miR-326, miR-26a) were identified as possible predictors for clinical resistance to imatinib in patients with newly diagnosed CML [13]. A relatively rapid increase in the expression of miR-150 and miR-146a and decrease of miR-142-3p and miR-199b-5p in peripheral blood mononuclear cells (PBMCs) of patients newly diagnosed with CML was found two weeks after imatinib initiation [14].

In this study, we used an array platform to characterize differentially expressed miRNAs in peripheral blood total leukocytes of patients at different stages of CML including diagnosis, major molecular response, therapy failure, hematological relapse, accelerated phase and blast crisis with the aim to identify microRNAs associated with pathogenesis of CML. To the best of our knowledge, such integrated microRNA profiling during the course of CML has not yet been performed. Hierarchical clustering analysis based on expression profiles of 49 miRNAs clearly separated patients at diagnosis, hematological relapse and blast crisis from those in major molecular response and therapy failure. We used *in silico *analyses to better understand the targets of 17 selected miRNAs whose deregulation was confirmed by real-time quantitative PCR (RT-qPCR). Based on our previous results demonstrating that miR-150 downregulation is associated with CML [15], we further validated miR-150 expression in a larger number of patients (n = 70). As *MYB *represents functionally validated target of miR-150 [16], its gene expression analysis was performed on the same patient cohort. Our data provide significant inverse correlations between miR-150 and *MYB *expression and *BCR-ABL *transcript level and indicate that this relationship is potentially important for pathogenesis in CML.

## Materials and methods

### Patient samples

Twenty four patient samples of total leukocytes from peripheral blood (Table 1) were used to prepare pools representing different CML phases for microarray analysis: diagnosis (n = 5, Dg), major molecular response (n = 5, MMR), therapy failure (n = 5, TF), hematological relapse (n = 5, Hr), and blast crisis (n = 4, BC). Briefly, Dg, Hr and BC contain 100% of Ph+ cells. Therapy failure is defined here as complete hematological response with failure to achieve complete cytogenetic remission (CCgR). Hematological relapse is defined as increased number of WBC (range 14-28*10^9^/L). MMR samples are characterized as *BCR-ABL *<0.1% (IS). BC samples contain blast cells in peripheral blood from 50% to 79%. Eleven healthy donors of age median 60 (range 45 - 78) and man/woman ratio 3/2 following CML incidence were used to create a control pool.

**Table 1:** Characteristics of patient samples in the pools

Pools	Patient number (gender)	IM therapy (months)	WBC count (*10^**9**^**/L PB)**	Thrombocyte count (*10^**9**^**/L PB)**	Blast count (% in PB)	BCR-ABL (%)	Ph+ cells (%)	Pretreatment	BCR-ABL mutations
**Dg**	1 (F)	0	71.4	749	0	60	100	HU	NA
	2 (F)	0	66.5	824	3	131	100	HU	NA
	3 (M)	0	22.3	509	1	144	100	HU	NA
	4 (M)	0	15.5	636	0	312	100	HU	NA
	5 (M)	0	198.5	550	1	139	100	HU, IFN	NA

**MMR**	6 (M)	14.8	5.7	244	0	0.001	0	HU	NA
	7 (F)	7.4	5.3	153	0	0.01	0	HU	NA
	8 (M)	9.2	6.1	181	0	0.001	0	HU	NA
	9 (M)	19.6	4.2	309	0	0.04	0	HU	NA
	10 (M)	13.1	4.8	230	0	0.02	0	HU	NA

**TF**	11 (F)	17.6	4.8	224	0	37	90	IFN	M351T
	12 (M)	14.3	4.2	142	0	15	100	HU	WT
	13 (F)	15.1	5.4	252	0	37	30	HU	WT
	14 (F)	13.5	2.4	100	0	23	60	HU, IFN	WT
	15 (M)	13.1	4.5	171	0	38	100	HU	WT

**Hr**	16 (M)	13.6	28.1	448	0	80	100	HU	M351T, F317L
	17 (F)	16.6	14.0	440	0	212	100	HU, IFN	F311I
	18 (M)	16.5	17.3	444	0	68	100	IFN, HU	M244V
	19 (F)	25.3	16.2	550	0	66	100	IFN	M351T
	20 (M)	22.4	26.1	245	0	164	100	IFN, HU	F317L

**BC**	21 (F)	55.4	65.6	147	62	1883	100	NA	M244V
	22 (M)	6.7	17.8	494	51	2188	100	NA	WT
	23 (M)	33.0	55.7	14	79	2500	100	NA	WT
	24 (M)	36.7	8.0	21	76	1000	100	NA	M351T, D276G

BC = blast crisis; Dg = Diagnosis; F = female; Hr = hematological relapse; HU = hydroxyurea; IF = interpheron alpha; IM = imatinib; M = male; MMR = major molecular response; PB = peripheral blood; TF = therapy failure; WBC = white blood cell, WT = wild type.

Seventy patient samples of total leukocytes from peripheral blood were used for miR-150 expression validation and *MYB *expression analyses (Table 2). Of these, 13 represented Dg, 16 = MMR, 14 = TF, 15 = Hr and 12 = AP (accelerated phase) together with BC (AP/BC). Therapy failure is defined here as non CCgR achievement; all patients achieved complete hematological remission and two patients major and minimal cytogenetic response, respectively. Hematological relapse is characterized by increased number of WBC and PLT (median 16*10^9^/L and 448*10^9^/L, respectively).

**Table 2:** Characteristics of patient samples in groups for miR-150 and *MYB *expression analysis

Disease stage	Number of patients	HU, IFN or combination pretreatment months from diagnosis median (range)	Months on imatinib median (range)	**WBC x10**^**9**^**/L PB median (range)** ** PLT x10**^**9**^**/L PB median (range)**	Blasts in PB (%) median (range)	Ph+ cells (%) median (range)	BCR-ABL (%) median (range)	BCR-ABL KD mutation Number of patients
**Dg**	13	NA	NA	67 (22-457)	2 (1-5)	100	132 (61-312)	NA
				481 (130-824)				

**AP/BC**	12	24 (2-106)	24 (8-55)	34 (1.17-147)	18 (8-76)	100	510 (103-2500)	3
				81 (14-562)				

**Hr**	15	18 (1-66)	22 (10-54)	16 (6-28)	10; 12	100 (32-100)	80 (23-827)	15
				448 (71-1779)				

**TF**	14	14 (1-68)	18 (12-67)	Phy	0	100 (40-100)	32 (11-91)	4
				Phy				

**MMR**	16	3 (1-11)	16 (7-24)	Phy	0	0	0.02 (0.001-0.1)	NA
				Phy				

AP = accelerated phase; BC = blast crisis; Dg = Diagnosis; Hr = hematological relapse; MMR = major molecular response; PB = peripheral blood; Phy = physiological (WBC 4-10 × 10^9^/L; PLT 150-400 × 10^9^/L ); PLT = platelets; TF = therapy failure; WBC = white blood cell.

The percentage of *BCR-ABL *transcript level was observed from the routine monitoring using real-time qPCR that is standardized within the frames of international standardization [17]. Mutation analyses were performed by direct sequencing method [18].

All subjects donated their samples with informed consent approved by the Ethic Committee of the Institute of Hematology and Blood Transfusion, Prague.

### Sample preparation

Cell pooling was applied for microarray analysis at the aim to reduce individual variability and to find common features of the disease stage. Pooling strategy was performed according to previously described recommendations [19,20]. Pools consisted of five patient samples; the blast crisis pool contained only four samples due to lack of appropriate material. Samples were selected for pooling according to their similar characteristics listed in Table 1. Each patient contributed to the pool by the same amount of leukocytes (10^7^).

### RNA extraction

Two different approaches were initially tested to extract total RNA containing small RNA molecules: acidic phenol-chloroform procedure and miRVana kit (Ambion^®^, Applied Biosystems, Foster City, CA, USA). The quality and quantity of RNA were evaluated using Agilent 2100 Bioanalyser (Agilent Technologies, Santa Clara, CA, USA) and spectrophotometer (NanoDrop Technologies, Wilmington, DE, USA), respectively. The miRVana kit and phenol-chloroform procedure gave comparable results with respect to RNA quality (RNA integrity numbers 7.8 - 9.1 and 8.5 - 9.0, respectively) and quantity (mean total RNA amounts 5 ug and 3 ug, respectively).

MiRVana kit however gave better 230/280 ratio and therefore was then selected for preparation of samples for microarrays. Acidic phenol-chloroform extraction was used for real-time qPCRs, which is a standardized method in our laboratory for *BCR-ABL *monitoring in international scale (IS).

### Microarray analysis

PIQOR™ miRXplore arrays (Miltenyi Biotech GmbH, Cologne, Germany) were used for miRNA expression profiling and the whole procedure including miRXplore data analysis (control vs. sample) was performed within the genomic facility of the manufacturer. Total RNAs with controlled quality (RIN 8 - 9.2; A260/A280 1.86 - 2.01; A230/A260 1.95 - 2.1) and quantity (1.2 - 4.0 μg) were sent to Miltenyi Biotech laboratory on dry ice. RNA quality and quantity was checked after delivery with the comparable results. Microarray platform contained 872 probes for human miRNAs according to miRBase version 10.1 and an extensive system of controls. Raw data were derived from ImaGene^® ^software (Biodiscovery, El Segundo, USA). Only spots with signal equal to or higher than 50% percentile of the background signal intensities were further analyzed.

The complete microarray data were deposited in Gene Expression Omnibus (GEO) database under the accession number GSE26260 (http://www.ncbi.nlm.nih.gov/geo/).

We applied MultiExperiment Viewer (MeV v4.0 release; http://www.tm4.org/mev) for k-means/medians and hierarchical clustering was performed using average linkage and average dot product metric.

### Real-time qPCRs

Real-time qPCR was performed on RotorGene 6000 (Qiagen, San Francisco, CA, USA). The miRNA expression assay kits (Applied Biosystems) specific for selected miRNAs were used to perform reverse-transcriptions and RT-qPCRs. MiR-30c showed stable expression across all the patient and control samples analyzed (stable Ct/ngRNA) and was used as a housekeeping gene for normalization. Relative fold changes of gene expression were assessed using 2^-ΔΔCT ^method. Mean of ΔCT values of 11 healthy donors was used as a calibrator. Results are presented as expression fold change of a patient to a healthy control.

TaqMan Gene Expression Assay (product number Hs00193527; Applied Biosystems) was used for *MYB *transcript quantification according to the manufacturer's recommendations. The *GUS *gene was used as the housekeeping gene with the primer set, probe, and protocol adopted from Beillard et al. [21].

### Prediction of putative miRNA target genes

The TargetScan Human release 5.1. (http://www.targetscan.org) was used for prediction of miRNA targets. Visualization and Integrated Discovery (DAVID) (http://david.abcc.ncifcrf.gov/) [22,23] was applied to annotate the biological functions of the predicted targets.

### Statistical analyses

Analyses of *MYB *and miR-150 differential expression between different groups of samples were conducted using Kruskall Wallis's test and Dunn's multiple comparison test. Correlation analyses were calculated using the Spearman's rho correlation test. Statistical analyses and graphs were performed using GraphPad Prison version 4.03 (GraphPad Software, La Jolla, CA, USA).

## Results

### miRNA expression profiles in CML

**Figure 1:**
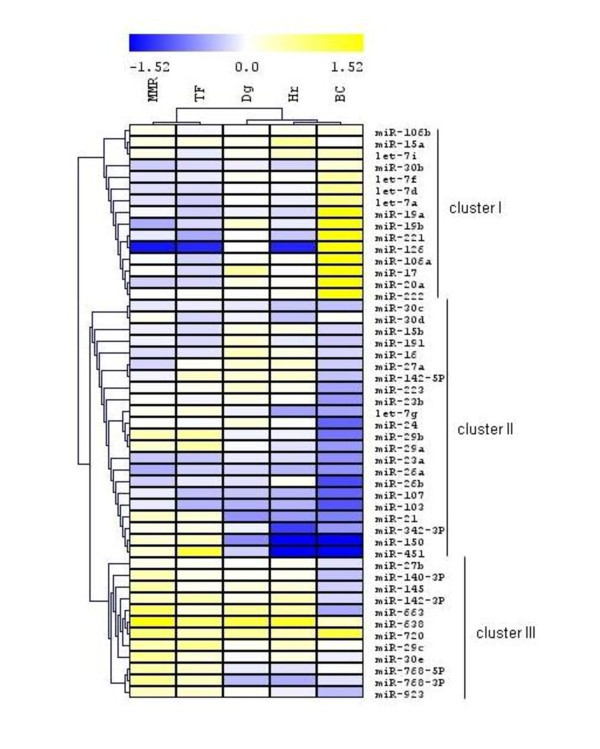
**Hierarchical clustering analysis of expression data of 49 microRNAs from microarray analysis**. Seven miRNAs are not displayed due to signal at the background level in at least one of the pools analyzed.

Microarray analysis in CML resulted in the detection of 56 differentially expressed miRNAs (samples vs. control). Figure 1 shows three main gene clusters (clusters I, II, III) of altogether 49 miRNAs. A markedly (more than 1.5 fold; others are not indicated here) increased level over the control was found in BC pool for miR-19a, miR-19b, miR-221, miR-126, miR-106a, miR-17, miR-20a and miR-222 that belong to the cluster I. A distant gene cluster II grouped down-regulated miRNAs in BC; more than 1.5 fold change was detected for miR-24, miR-29b, miR-26b, miR-107, miR-103, miR-150, miR-451. The cluster III consisted of miRNAs with increased level in MMR; more than 1.5-fold change was found for miR-663, miR-638 and miR-720.

A separate cluster was created from MMR and TF pools and was distant from the cluster grouping Dg pool together with Hr and BC pools. MMR and TF pools are represented by the samples from patients during the imatinib treatment with optimal response and failure to achieve CCgR, respectively. The samples from MMR and TF pools were characterized by physiological blood count but different *BCR-ABL *transcript level and number of Ph+ metaphases (Table 1). The distant cluster of Hr, BC and Dg pools represented CML in progression and at diagnosis, respectively.

### Validation of array data and sample pooling

**Figure 2:**
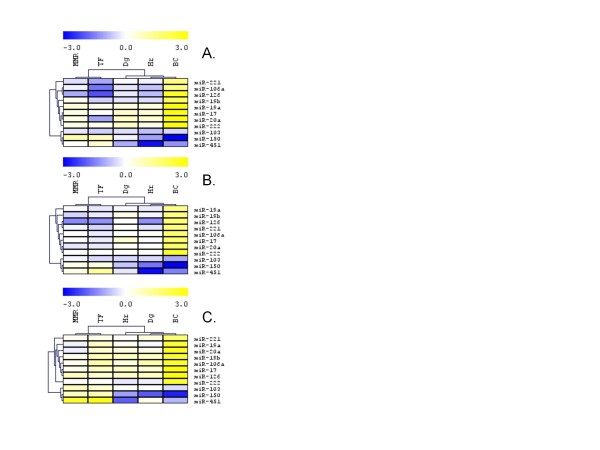
**Hierarchical clustering analysis of miRNA expression data**. (A.) microarray - pools, (B.) real-time qPCR - pools and (C.) real-time qPCR - individual samples.

**Figure 3:**
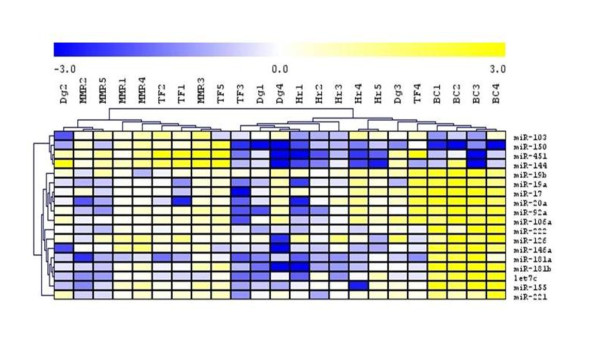
**Hierarchical clustering analysis of detected levels of 17 selected miRNAs by real-time qPCRs**.

Eight up-regulated (miR-19a, miR-19b, miR-221, miR-222, miR-126, miR-106a, miR-17 and miR-20a) and 3 down-regulated miRNAs (miR-103, miR-150 and miR-451) with more than 2.0-fold change in their expression (see Figure 1) over the control were selected for array data validation and for evaluation of pooling precision by the RT-qPCR. MiRNAs expressions were measured in the pools and in individual samples of each pool. The heatmaps showed comparable results (Figure 2); on comparing array and RT-qPCR data of pools and averaged RT-qPCR data of individuals, hierarchical clustering formed similar gene and sample clusters. We noted also few discrepancies e.g. for miR-126 and miR-451. We evaluated RT-qPCR data of each sample using hierarchical clustering (Figure 3). Seven miRNAs (miR-181a, miR-181b, miR-92a, miR-146a, let7c, miR-144, miR-155) that were not displayed in Figure 1 and 2 due to low signal on the array analysis in at least one of the pools, were included into the RT-qPCR analysis because of strong change in their expressions in BC pool. Three prominent patient clusters and three prominent miRNA clusters were identified (Figure 3). Firstly, a gene cluster distant from the other two consisted of miR-103, miR-150, miR-451 and miR-144. These molecules showed a rather decreased level at Dg, in Hr and BC. The other two closely related clusters consisted of miR-19b, miR-19a, miR-17, miR-20a, miR-92a, miR-106a, miR-222, miR-126, miR-146a, miR-181a, miR-181b, let7c, miR-155 and miR-221 that were up-regulated in BC samples.

The BC samples formed one cluster that was mixed with two samples from hematological relapse (Hr 4, 5), one from diagnosis (Dg 3) and one therapy failure (TF 4). This cluster was related to the cluster that was formed by Hr samples (Hr 1-3), two Dg samples (Dg 1, 4) and one therapy failure (TF 3). The third cluster distant from the other two consisted of all MMR samples, three TFs (TF 1, 2, 5) and was mixed with Dg 2 sample.

### Target in silico analyses and functional annotation

The putative target genes were selected for 17 miRNAs with validated expression in different phases of CML (Figure 3). The predicted targets containing highly conserved sites were further studied according to the P_CT _values [24] from the TargetScan release 5.1 except for miR-106a because of lack data in the database. Because the P_CT _values are available only in the TargetScan we did not use another databases for miR-106a target prediction to preserve data consistency.

The targets were selected according to P_CT _equal or higher than 0.1 and 0.5 of miRNAs with low and high number of targets in the database, respectively (see Additional file 1: Table S1). The P_CT_, ranging between 0 and 1, corresponds to a Bayesian estimate of the probability that a site conserved to a particular branch length is conserved due to miRNA targeting [24]. The 30% of all of the targets with only conserved sites (Additional file 1: Table S1) are putative target genes of more than one of the selected miRNAs.

Functional annotation analysis of predicted targets (Table 3) revealed several biological processes (P < 0.0001). The encoded proteins are involved mainly in the regulation of transcription, intracellular signaling cascades, amino acid phosphorylation, regulation of RNA metabolic processes, regulation of apoptosis, regulation of cell proliferation and protein transport. Several proteins are implicated in hematopoietic or lymphoid organ development (n = 52; e.g. BMI1, WNT3A, MLL5, IL25, CDK6, MYH9, BCL2L11, CRKL, KIT, BCL2, RUNX1, TCF3, PIK3R1, NOTCH2, PKNOX1, SP1, SP3, TGFBR3), regulation of erythrocyte differentiation (ACVR2A, ACVR1B, ETS1, MAFB, SPI1, CDK6, FOXO3, INPP5D, ARNT) and regulation of myeloid cell differentiation (ZFP36, HMGB3, MAFB, KLF10, NDFIP1, SPI1, CDK6, FOXO3, PRDM16, PURB, ARNT, LIF, ACVR1B, ACVR2A, ID2, ETS1, GNAS, INPP5D, RUNX1).

**Table 3:** Functional annotation of predicted targets

GO category	Count	% *	P-value
*Biological process*			
Regulation of transcription	407	22.0	4.28E-20
Intracellular signaling cascade	208	11.24	5.52E-12
Protein amino acid phosphorylation	127	6.86	2.03E-11
Regulation of RNA metabolic process	269	14.54	5.67E-10
Negative regulation of cellular biosynthetic process	107	5.78	8.68E-10
Phosphate metabolic process	159	8.59	7.5E-9
Regulation of small GTPase mediated signal transduction	58	3.14	1.41E-8
Regulation of Ras protein signal transduction	51	2.8	1.89E-8
Regulation of apoptosis	132	7.1	1.35E-7
Vesicle-mediated transport	101	5.5	2.2E-7
Negative regulation of signal transduction	50	2.7	2.77E-7
Protein transport	124	6.7	5.48E-7
Transmembrane receptor protein tyrosine kinase signaling pathway	49	2.65	1.04E-6
Regulation of cell migration	40	2.16	1.57E-6
Positive regulation of cell differentiation	49	2.65	2.03E-6
Intracellular transport	108	5.84	2.04E-6
Regulation of cell proliferation	124	6.7	2.99E-6
Hematopoietic or lymphoid organ development	52	2.8	7.41E-6
Regulation of protein kinase cascade	47	2.5	9.5E-5
Regulation of protein kinase activity	60	3.24	1.05E-4
Regulation of erythrocyte differentiation	9	0.5	2.41E-4
Regulation of myeloid cell differentiation	19	1.2	2.42E-4
			
*Molecular function*			
Transcription regulator activity	268	14.5	8.81E-19
GTPase regulator activity	82	4.4	1.32E-8
Protein kinase activity	110	5.95	2.33E-8
Cytoskeletal protein binding	90	4.86	1.06E-6
Transcription repressor activity	63	3.41	1.46E-6
Protein domain specific binding	65	3.51	1.67E-6
DNA binding	314	16.97	2.51E-6
Zinc ion binding	303	16.38	3.7E-5
Chromatin binding	34	1.84	4.03E-5
Cation binding	509	27.51	1.28E-4
SH3 domain binding	24	1.3	1.51E-4
			
*Pathway*			
Endocytosis	45	2.43	1.93E-8
Pathways in cancer	64	3.46	1.41E-7
mTOR signaling pathway	16	0.86	1.13E-4
Hedgehog signaling pathway	16	0.86	2.82E-4
Chronic myeloid leukemia	19	1.03	3.21E-4
Focal adhesion	37	2.0	3.39E-4
Wnt signaling pathway	29	1.57	8.77E-4

* from total 1850

Major Gene Ontology (GO) categories. The threshold of statistical significance for GO enrichment in the gene list was set up to P ≤ 10^-4^.

Using KEGG database [25] we analyzed signaling pathways with significant hits (P < 0.0001) for predicted targets involved in endocytosis (hsa04144), pathways in cancer (hsa05200), mTOR signaling pathway (hsa04150), hedgehog signaling pathway (hsa04340), chronic myeloid leukemia (hsa05220), focal adhesion (hsa04510) and Wnt signaling (hsa04310) (Table 3).

Table 4 summarizes predicted targets associated with chronic myeloid leukemia. Most of them are involved in MAPK signaling (BCR, E2F2, E2F3, CBL, RAF1, CRK, CRKL, KRAS, SOS1, MAPK1). TGBR2, SMAD4 and ACVR1B play a role in transforming growth factor beta signaling pathway. Cyclin D1 (CCND1) and cyclin-dependent kinase 6 (CDK6) are important for the cell cycle and in the p53 pathway. Cell cycle is influenced by cyclin-dependent kinase inhibitor 1B (p27). ErbB signaling pathway encompasses PIK3R1 and PIK3R3. RUNX1 known as AML1 contributed to the abnormality in growth inhibition.

**Table 4:** Target annotation in pathways of chronic myeloid leukemia (hsa05220)

microRNAs	Targets*	Definition	**P**_**CT**_	Pathway in CML
miR-20a	BCR	Breakpoint cluster region protein	0.56	
	
miR-222	E2F2	E2F transcription factor 2	0.32	
miR-17	E2F2		0.59	
miR-155	E2F2		0.78	
	
miR-17	E2F3	E2F transcription factor 3	0.54	
	
miR-150	CBL	E3 ubiquitin-protein ligase	0.45	
miR-222	CBL		0.33	
miR-155	CBL		0.67	
	
miR-19a	RAF1	RAF proto-oncogene serine/threonine-protein kinase	0.82	**MAPK signaling **
				**pathway**
miR-126	CRK	Proto-oncogene C-crk	0.55	
miR-17	CRK		0.88	
	
miR-221	CRKL	Proto-oncogene C-crk	0.16	
	
miR-19a	KRAS	GTPase	0.92	
miR-155	KRAS		0.33	
	
miR-155	SOS1	Son of sevenless	0.53	
miR-181a	SOS1		0.78	
	
miR-19a	MAPK1	Extracellular signal-regulated	0.86	
miR-17	MAPK1	kinase 1/2 (ERK)	0.96	

miR-19a	TGFBR2	Transforming growth factor -beta	0.87	
miR-144	TGFBR2	receptor type-2	0.65	
miR-155	TGFBR2		0.28	**Transforming growth factor ß**
				**signaling pathway**
miR-144	SMAD4	Mothers against DPP homolog 4	0.58	
	
miR-17	ACVR1B	Transforming growth factor -beta receptor type-1	0.52	

miR-19	CCND1	Cyclin D1	0.86	
miR-17	CCND1		0.97	**p53 pathway**
miR-155	CCND1		0.55	
				**Cell cycle**
miR-103	CDK6	Cyclin-dependent kinase 6	0.73	

miR-222	CDKN1B	Cyclin-dependent kinase inhibitor 1B (p27)	0.50	**Cell cycle**

miR-103	PIK3R1	Phosphoinositide-3-kinase,	0.73	
miR-221	PIK3R1	regulatory subunit	0.63	
miR-155	PIK3R1		0.28	**ErbB signaling**
				**pathway**
miR-19a	PIK3R3	Phosphoinositide-3-kinase,	0.92	
miR181a	PIK3R3	regulatory subunit	0.81	

miR-17	RUNX1	Runt-related transcription factor 1	0.88	**Abnormality in**
miR-144	RUNX1	(AML1)	0.59	**growth inhibition**
				

* official gene symbol

### MiR-150 down-regulation and targeted MYB overexpression

**Figure 4:**
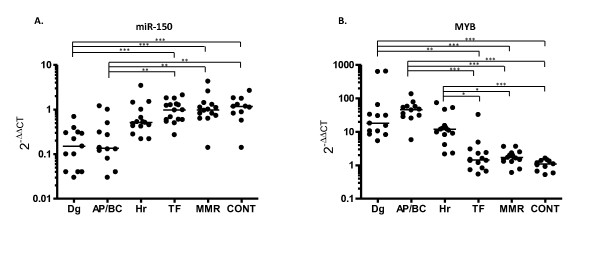
**Expression analysis of miR-150 (A.) and its target *MYB *(B.) in different phases of CML in comparison to control (CONT)**. CONT = 11, Dg = 13 diagnosis, AP/BC = 12 accelerated phase/blast crisis, Hr = 15 hematological relapse, TF = 14 therapy failure (failure to achieve complete cytogenetic remission), MMR = 16 major molecular response. *** P < 0.001;	 ** P < 0.01; * P < 0.05

Dramatic reduction of miR-150 in BC, at Dg and in Hr and its normal levels in patients under imatinib treatment (MMR and TF) (Figure 3) prompted us to determine this expression pattern on a larger cohort of patients (n = 70; Table 2). Significant down-regulation of the miRNA (p < 0.05) in comparison to healthy controls (n = 11) was confirmed for diagnosis and progressed phases of CML (Figure 4A.). MiR-150 level decreased more than 2-fold in 67% of hematological relapses (n = 10/15). There was no significant change in MMR and TF compared to controls. Among all patient samples analyzed, we found significant inverse correlation of miR-150 expression with *BCR-ABL *transcript level (p = 0.01; r = -0.501). To test whether miR-150 is regulated by BCR-ABL we have used a Ph+ cell line MOLM-7 and incubated it with two concentrations of imatinib (1uM and 10 uM, Additional file 2: Figure S1A) for total 48 hours. We observed that following reduction of BCR-ABL tyrosine kinase activity (exemplified by decreased intensity of p-CRKL (Additional file 2: Figure S1B)) by imatinib the miR-150 levels were significantly upregulated. This paragraph provides link between levels of one particular microRNA, miR-150, identified by our microarray analysis and CML pathogenesis.

*MYB *is a confirmed target of miR-150 [16]. The expression pattern of *MYB *during the course of CML has not as yet been reported. We decided to analyze *MYB *transcript levels in the same cohort of patients. *MYB *was significantly increased at Dg, in AP/BC and Hr in comparison to controls (p < 0.001), to MMR (p < 0.05) and to TF (p < 0.05). Spearman's rho analysis displayed significant inverse correlation of *MYB *with miR-150 expression (p = 0.01; r = -0.521) and significant positive correlation between *MYB *expression and *BCR-ABL *transcript level (p = 0.01; r = 0.771).

## Discussion

Specific microRNAs regulate hematopoietic cell differentiation and development [26]. The main interest is in whether there exists a link between levels of miRNAs and leukemia pathogenesis. The first work dealing with miRNA expression in CML demonstrated enhanced expression of the miR-17-92 cluster in CML CD34+ cells [11]. Other works that reported miRNA aberrant expression in CML appeared very recently. For example, it demonstrated that several miRNAs dysregulated in CML (miR-150, miR-146a, miR-142-3p, miR-199b-5p) were rapidly restored under imatinib treatment [14]. Several miRNAs (e.g. miR-191, miR-29a, miR-422b, miR-100, miR-326, miR-26a) are promising predictors of imatinib resistance in newly diagnosed CML patients [13].

This study investigates microRNA differential expression profiles that were initially analyzed at different stages of CML using microarrays. Pooling of patient samples was applied for microarray analysis to reduce individual variability and to find common features of the disease.

MiRNA array data showed similar expression pattern of 49 miRNAs in imatinib responders with MMR and patients with failure to achieve complete cytogenetic response. As expected, hierarchical clustering assembled the pools of samples at diagnosis, in hematological relapse and blast crisis, while MMR and TF pools formed a separate cluster (Figure 1). Total leukocytes from blast crisis peripheral blood that consisted of more than 50% blasts of each sample in the pool showed the highest number of strongly deregulated miRNAs.

We applied the functional annotation tool DAVID to look for the biological functions of predicted targets with only conserved sites and high P_CT _values of the 17 miRNAs with real-time qPCR-confirmed up-regulation (miR-19a, miR-19b, miR-17, miR-20a, miR-92a, miR-221, miR-222, miR-126, miR-146a, miR-181a, miR-181b, let7c and miR-155) and down-regulation (miR-103, miR-150, miR-451 and miR-144) in blast crisis (Figure 3). Several targets were involved in the processes that were found to be important in CML; endocytosis [27], mTOR signaling pathway [28,29], hedgehog signaling [30,31], focal adhesion [32,33] and Wnt signaling [34,35]. We summarized 19 genes with the probability to be targeted by miR-20a, miR-17, miR-19a, miR-103, miR-144, miR-150, miR-155, miR-181a, miR-221 and miR-222. The encoded proteins were annotated in pathways related to the CML (hsa05220). Out of these, 10 targets are involved in MAPK signaling (BCR, E2F2, E2F3, CBL, RAF1, CRK, CRKL, KRAS, SOS1, MAPK1). Interestingly, inhibition of MAPK signaling in Ph+ cell line K562 induced apoptosis [36]. Application of MAPK specific inhibitor U0126 showed synergistic effect with imatinib resulting in CD34+ progenitor reduction in CML [36].

Confirmed increase of miR-19a, miR-19b, miR-17, miR- 20a, miR-92a, miR-106a, miR-221, miR-222, miR-126, miR-146a, miR-181a, miR-181b, let7c and miR-155 was identified in samples of BC pool (Figure 3). This pattern was not found in Dg, Hr, TF or MMR pools. Overexpression of these miRs may be related to the immature character of blasts. Whether the increased level of these miRNAs may contribute to the CML pathogenesis or may simply reflect the stage of the disease is the matter of further investigation. Abnormal expression of onkomir miR-17-92 (miR-17; miR-19a; miR-19b; miR-20a; miR-92a) was described in CML CD34+ cells [11]. Agirre et al. [12] found up-regulated miR-221 and miR-222 in mononuclear cells of CML patients in comparison to healthy controls. MiR-155, miR-106a, miR-146a, miR-181 and miR-126 were reported as deregulated miRNAs in CML [13,14]. To our knowledge, let7c expression has so far not been described in CML. In this study, our *in silico *analyses revealed that miR-221 and miR-103 (P_CT _0.63 and 0.73, respectively) target PIK3R1. PIK3R3 is predicted to be regulated by miR-19a and miR-181a (P_CT _0.92 and 0.81, respectively). PI3K is annotated in ERBB, MAPK and mTOR signaling pathways. KRAS, which is involved in MAPK signaling, is a predicted target of miR-19a (P_CT _0.92). MAPK expression may be regulated by onkomirs miR-17 and miR-19a (P_CT _0.96 and 0.86, respectively). Interestingly, it was reported that RAS/MAPK signaling may contribute to the survival of BCR-ABL positive cells under imatinib selection pressure [37]. AKT1, a member of the antiapoptotic PI3K pathway, is involved in both, BCR-ABL mediated transformation as well as in response to the BCR-ABL kinase inhibitors. It was shown that the PI3K/AKT/mTOR signaling is activated in imatinib naive cells while under imatinib pressure it may enhance resistance to imatinib [38]. As shown in our real-time qPCR data (Figure 3), the rather decreased levels of miR-181a, miR-221 and miR-19a in some imatinib treated patients, and miR-103 down-regulation in a number of blast crisis, diagnosis and progressed CML may contribute to the increased level of PI3K and thus may be involved in the previously described PI3K/AKT/mTOR signaling activation and in the resistance development in some CML cases. Though no experimental therapy using miRNA modulation has as yet provided significant and curative approach, the knowledge of deregulation of miRNAs specific for CML may facilitate the development of such therapeutic strategies. Several candidate microRNAs (e.g. miR-181a, miR-221, miR-19a, miR-103) regulating expression in CML target important signaling pathways may represent promising candidate targets for CML therapy.

The real-time qPCR validated the down-regulation of miR-150, miR-451, miR-103 and miR-144 overall in individual samples of BC, Hr, Dg pools and in some samples of TF pool (Figure 3). These molecules may be related to the CML pathogenesis and may reflect transformation from chronic to accelerated phases. Agirre et al. found miR-150 downregulation in mononuclear cells and CD34+ cells separated from bone marrow in newly diagnosed CML patients (n = 6) in comparison to healthy donors (n = 6) [12]. MiR-150 was recently described to be downregulated in untreated CML patients [14]. Flamant et al. [14] suggest that miR-150 play a role in leukemic cells and potentially in the more primitive hematopoietic compartment in chronic phase CML patients. This is in line with the knowledge that miR-150 is important in the regulation of hematopoiesis. During normal erythroid differentiation its level is gradually decreased [39], however; it shows the highest expression in mature lymphocytes [40]. Others proved that miR-150 expression increases during B-lymphoid differentiation in contrast to myeloid differentiation. It seems likely that miR-150 regulates the development of other two different blood lineages; B lymphocytes and megakaryocytes [41,42]. Thus, miR-150 deregulation is found in hematological malignancies; miR-150 is decreased in polycytemia vera reticulocytes [43] and a marked decrease was recently also detected in MDS-del(5q) [44] while, in contrast, a twofold increase was found in CLL lymphocytes [45].

Based on our results [15] and recent results of others we expanded real-time qPCR assays of miR-150 on the larger cohort of CML patients. Decreased level of miR-150 was confirmed in patients at diagnosis, in the majority of patients with hematological relapse and in accelerated phase and blast crisis. Normal miR-150 level was observed in imatinib treated patients with major molecular response and failure to achieve CCgR. Our observations are consistent with the data of Flamant et al. [14] showing rapid increase of miR-150 expression after imatinib treatment initiation in patients with newly diagnosed CML. They further found that low miR-150 expression inversely correlated with white blood count and thus speculated that the level reflected the high leukocyte counts in newly diagnosed CML patients. We showed here a significant inverse correlation of miR-150 expression with *BCR-ABL *transcript level (p = 0.01; r = -0.501). Non-treated newly diagnosed patients, patients with disease progression and resistant to imatinib showed a high level of *BCR-ABL *together with high leukocyte count and decreased amount of miR-150. Normal miR-150 level was detected in imatinib responders (MMR) and patients with failure to achieve CCgR (TF) with normal blood count and low *BCR-ABL *transcript level. As imatinib targets Ph+ cells, the normal level of miR-150 in imatinib treated patients in chronic phase with physiological blood count could be the result of the suppression of leukemic cells and the concomitant recovery of normal hematopoiesis under imatinib treatment. Our *in vitro *tests showed elevated expression of miR-150 and marked decrease of p-CRKL following imatinib *in vitro *treatment of Ph+ cell line MOLM-7. These findings suggest a potential functional relationship between miR-150 and BCR-ABL.

Gene expression of *MYB *in our study showed a significant inverse correlation with miR-150 transcript level (p = 0.01; r = -0,409). *MYB *is the proven target of miR-150 and encodes a transcriptional factor required for proliferation and survival of normal and leukemic blast cells. A recently published study on a mouse model of blast crisis reported that *c-MYB *is required for BCR-ABL dependent leukemogenesis [46]. Lidonnici et al. [46] speculated that miR-150 reduction might contribute to the *c-MYB *upregulation that is likely induced by BCR-ABL, and may be involved in BCR-ABL driven leukemogenesis in CML. Interestingly, we found a significant correlation between *MYB *expression and *BCR-ABL *transcript level (p = 0.01; r = 0,782) in CML patients, which is in line with the above described suggestion.

In summary, our data demonstrated that miR-150, miR-20a, miR-17, miR-19a, miR-103, miR-144, miR-155, miR-181a, miR-221 and miR-222 are deregulated in CML. Furthermore, *in silico *filtering identified targeted genes that are involved in cell cycle, growth inhibition, MAPK, ErBb, transforming growth factor beta and p53 signaling pathways that are reported in CML pathogenesis. MiR-150 expression showed significant negative correlation with its target *MYB *and with *BCR-ABL *transcript level. The results of this study outline the mechanisms whereby miRNAs may be implicated in CML pathogenesis. However, if they function in BCR-ABL dependent or independent manner has to be elucidated.

## Competing interests

The authors declare that they have no competing interests.

## Authors' contributions

KMP - conception and design, *in silico *analyses, data evaluation and interpretation, manuscript drafting; TL - contribution to the manuscript drafting, real-time qPCR analyses and evaluation; HK - provision of patient samples, clinical data evaluation, critical revisions; PB - *in vitro *tests, contribution to data evaluation and interpretation, critical revision; MT - clinical data revision; critical revisions of the manuscript; TS - contribution to data evaluation and interpretation, critical revision; JM - critical revisions. All authors read and approved the final manuscript.

## Supplementary Material

Additional file 1 **Table S1: Number of predicted targets of conserved miRNA families**. Table summarizes number of the microRNA targets that were selected according to P_CT _equal or higher than 0.1 and 0.5. Click here for file

Additional file 2 **Figure S1: MiR-150 exression is elevated and BCR-ABL activity is dropped after imatinib treatment in Ph+ MOML-7 cells**. Data from *in vitro *test of miR-150 expression change after Ph+ cell line incubation with imatinib. (A.) MiR-150 expression change after imatinib treatment in Ph+ MOML-7 cells. 3 × 10^6 ^MOLM-7 cells were incubated for 24 or 48hrs with or without (white bars; CTRL = control) imatinib. Two different concentrations of imatinib were tested (1 μM - gray bars and 10 μM - black). Cellular RNA was isolated by Trizol (Invitrogen), transcribed using High Capacity cDNA Reverse Transcription Kit (Roche Diagnostics). Real-time qPCR was performed using TaqMan protocol (Roche Diagnostics) and was run on the ABI 7900HT instrument. RNU44 was used as housekeeping gene. Data were evaluated by 2^-ΔCt ^method. The viability of culture with imatinib decreased: 1 μM imatinib- from 94% after 24hrs to 21% after 48hrs; 10 μM imatinib- from 95% after 24hrs to 18% after 48hrs. (B.) The intensity change of p-CRKL after imatinib treatment in Ph+ MOLM-7 cells. The amount of p-CRKL (a client molecule of BCR-ABL tyrosine kinase) was measured by standard western blot analysis (p-CRKL (Tyr207) antibody; Cell Signaling Technology) after 24h culture cultivation with imatinib using both concentrations. Beta-actin (monoclonal antibody Anti-beta-Actin, Sigma) was used as the loading control and was measured by western blot analysis using alkaline phosphatase. The measurement of p-CRKL was not possible to perform in the culture after 48 incubation due to a marked viability decrease and thus to low amount of material. Click here for file
