# Supplementary material for: Expression patterns of microRNAs associated with CML phases and their disease related targets
Source: Mol Cancer. 2011 Apr 18;10:41. doi: 10.1186/1476-4598-10-41 (PMC3102634; doi:10.1186/1476-4598-10-41)
Supplement: Additional file 1 — Table S1: Number of predicted targets of conserved miRNA families. Table summarizes number of the microRNA targets that were selected according to PCT equal or higher than 0.1 and 0.5. [file 1476-4598-10-41-S1.DOC]

| **microRNA**  **conserved families** | **P_CT_** | **Number of targets** |
| --- | --- | --- |
| mir-150 | ≥0.1 | 46 |
| mir-451 | ≥0.1 | 6 |
| mir-103 | ≥0.5 | 308 |
| mir-155 | ≥0.5 | 150 |
| mir-221/222 | ≥0.5 | 297 |
| mir-126 | ≥0.1 | 14 |
| mir-19a/19b | ≥0.5 | 908 |
| mir-17/20a | ≥0.5 | 525 |
| mir-144 | ≥0.5 | 621 |
| mir-181a/b | ≥0.5 | 262 |
| mir-146a | ≥0.1 | 22 |
| let7c | ≥0.1 | 23 |
| miR-92a | ≥0.1 | 31 |
|  |  |  |
