# Supplementary material for: Expression patterns of microRNAs associated with CML phases and their disease related targets
Source: Mol Cancer. 2011 Apr 18;10:41. doi: 10.1186/1476-4598-10-41 (PMC3102634; doi:10.1186/1476-4598-10-41)
Supplement: Additional file 2 — Figure S1: MiR-150 exression is elevated and BCR-ABL activity is dropped after imatinib treatment in Ph+ MOML-7 cells. Data from in vitro test of miR-150 expression change after Ph+ cell line incubation with imatinib. (A.) MiR-150 expression change after imatinib treatment in Ph+ MOML-7 cells. 3 × 10^6^ MOLM-7 cells were incubated for 24 or 48hrs with or without (white bars; CTRL = control) imatinib. Two different concentrations of imatinib were tested (1 μM - gray bars and 10 μM - black). Cellular RNA was isolated by Trizol (Invitrogen), transcribed using High Capacity cDNA Reverse Transcription Kit (Roche Diagnostics). Real-time qPCR was performed using TaqMan protocol (Roche Diagnostics) and was run on the ABI 7900HT instrument. RNU44 was used as housekeeping gene. Data were evaluated by 2-ΔCt method. The viability of culture with imatinib decreased: 1 μM imatinib- from 94% after 24hrs to 21% after 48hrs; 10 μM imatinib- from 95% after 24hrs to 18% after 48hrs. (B.) The intensity change of p-CRKL after imatinib treatment in Ph+ MOLM-7 cells. The amount of p-CRKL (a client molecule of BCR-ABL tyrosine kinase) was measured by standard western blot analysis (p-CRKL (Tyr207) antibody; Cell Signaling Technology) after 24h culture cultivation with imatinib using both concentrations. Beta-actin (monoclonal antibody Anti-beta-Actin, Sigma) was used as the loading control and was measured by western blot analysis using alkaline phosphatase. The measurement of p-CRKL was not possible to perform in the culture after 48 incubation due to a marked viability decrease and thus to low amount of material. [file 1476-4598-10-41-S2.PPT]

## Slide 1
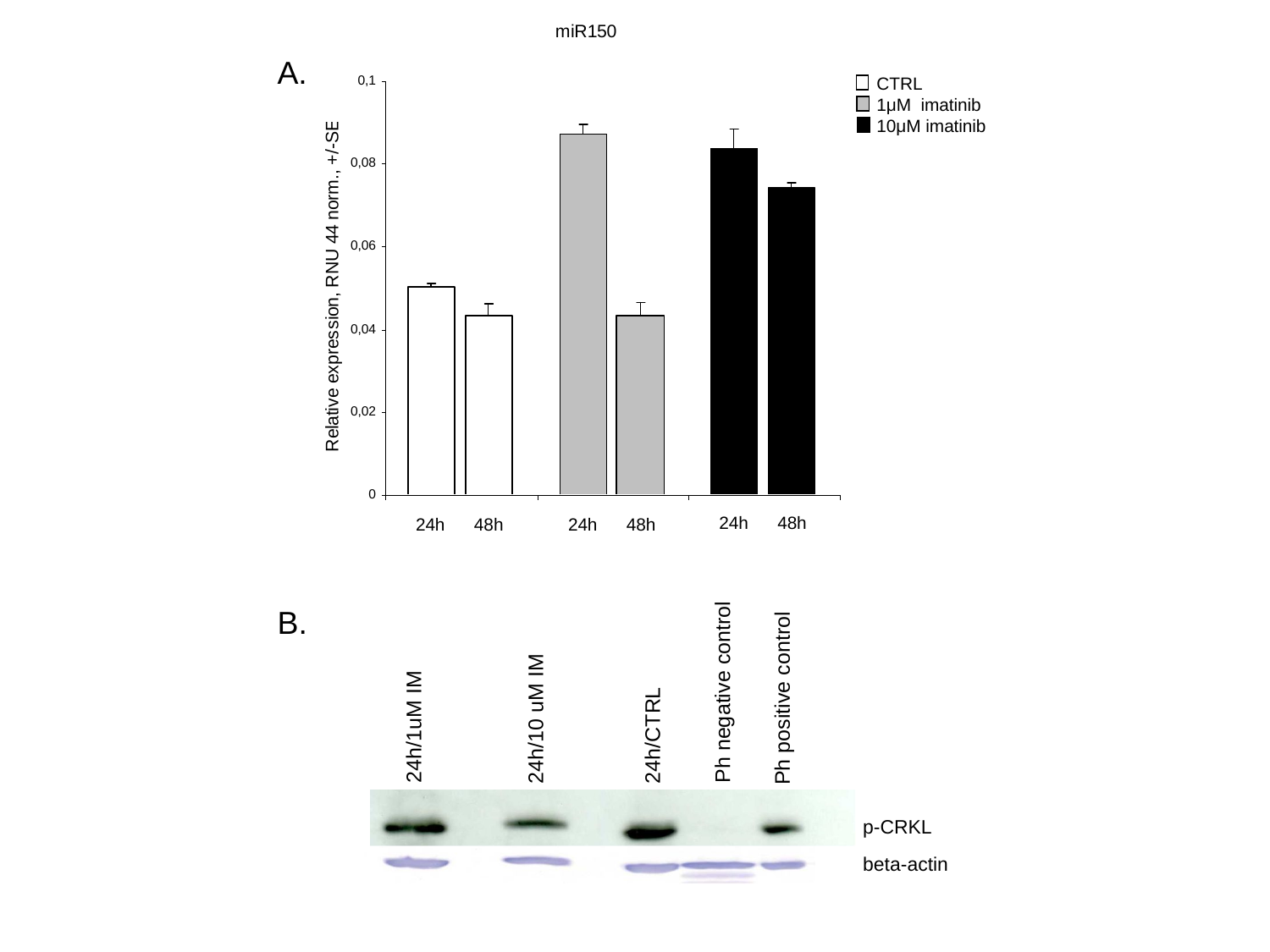

A.
CTRL
1μM imatinib
10μM imatinib
24h/10 uM IM
24h/CTRL
24h/1uM IM
p-CRKL
beta-actin
Ph negative control
Ph positive control
24h 48h
24h 48h
24h 48h
B.
